# Sensitivity Analyses for Robust Causal Inference from Mendelian Randomization Analyses with Multiple Genetic Variants

**DOI:** 10.1097/EDE.0000000000000559

**Published:** 2016-11-30

**Authors:** Stephen Burgess, Jack Bowden, Tove Fall, Erik Ingelsson, Simon G. Thompson

**Affiliations:** From the a Cardiovascular Epidemiology Unit, Department of Public Health and Primary Care, University of Cambridge, Cambridge, United Kingdom; b Medical Research Council Integrative Epidemiology Unit, School of Social and Community Medicine, University of Bristol, Bristol, United Kingdom; and c Department of Medical Sciences, Molecular Epidemiology, Uppsala University, Uppsala, Sweden.

## Abstract

Mendelian randomization investigations are becoming more powerful and simpler to perform, due to the increasing size and coverage of genome-wide association studies and the increasing availability of summarized data on genetic associations with risk factors and disease outcomes. However, when using multiple genetic variants from different gene regions in a Mendelian randomization analysis, it is highly implausible that all the genetic variants satisfy the instrumental variable assumptions. This means that a simple instrumental variable analysis alone should not be relied on to give a causal conclusion. In this article, we discuss a range of sensitivity analyses that will either support or question the validity of causal inference from a Mendelian randomization analysis with multiple genetic variants. We focus on sensitivity analyses of greatest practical relevance for ensuring robust causal inferences, and those that can be undertaken using summarized data. Aside from cases in which the justification of the instrumental variable assumptions is supported by strong biological understanding, a Mendelian randomization analysis in which no assessment of the robustness of the findings to violations of the instrumental variable assumptions has been made should be viewed as speculative and incomplete. In particular, Mendelian randomization investigations with large numbers of genetic variants without such sensitivity analyses should be treated with skepticism.

## Abstract 2

Supplemental Digital Content is available in the text.

An instrumental variable in an observational study behaves similarly to random treatment assignment in an experimental setting.^1^ It provides a natural experiment, whereby individuals with different levels of the instrumental variable differ on average with respect to the putative risk factor, but not with respect to any confounders of the risk factor–outcome association.^2^ Mendelian randomization is the use of a genetic variant as a proxy for a modifiable risk factor.^3,4^ If a genetic variant satisfies the assumptions of an instrumental variable for the risk factor, then whether there is an association between the genetic variant and the outcome is a test of whether the risk factor is a cause of the outcome.^5^

The instrumental variable assumptions are satisfied for a genetic variant if

(i) the genetic variant is associated with the risk factor;
(ii) the genetic variant is not associated with confounders of the risk factor–outcome relationship; and
(iii) the genetic variant is not associated with the outcome conditional on the risk factor and confounders of the risk factor–outcome relationship.^6^

These assumptions imply that the only causal pathway from the genetic variant to the outcome is via the risk factor, and there is no other causal pathway either directly to the outcome or via a confounder.^7^ A diagram corresponding to these assumptions is presented in Figure 1.

**FIGURE 1.**
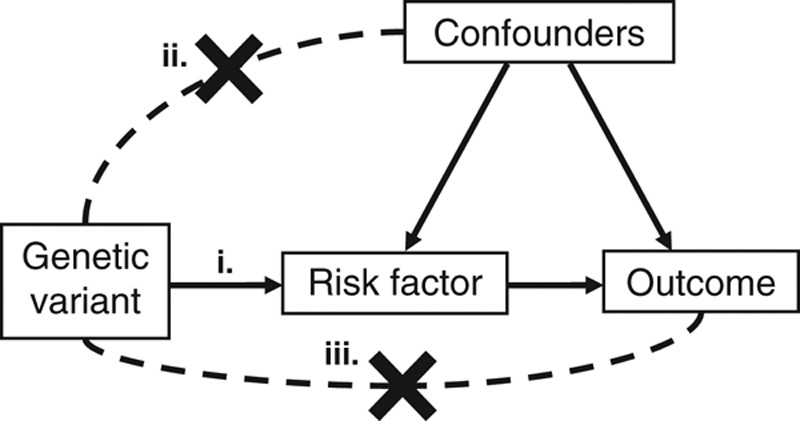
Diagram of instrumental variable assumptions for Mendelian randomization. The three assumptions (i, ii, iii) are illustrated by the presence of an arrow, indicating the effect of one variable on the other (assumption i), or by a dashed line with a cross, indicating that there is no direct effect of one variable on the other (assumptions ii and iii).

We further assume that all valid instrumental variables identify the same causal parameter; we return to this assumption in the discussion. For this interpretation to hold, it is necessary for certain parametric assumptions to hold. In this article, we assume that the effects of (i) the instrumental variables on the risk factor, (ii) the instrumental variables on the outcome, (iii) the risk factor on the outcome are linear without effect modification; and (iv) the association of the genetic variant with the risk factor is homogeneous in the population.^5^ These assumptions are not necessary for the identification of a causal effect, but they ensure that the estimate from each instrumental variable targets the same average causal effect.^8^ Weaker assumptions can identify a local average causal effect;^9^ however, the local average causal effect is likely to differ for each instrumental variable. Although these assumptions are strict, the causal estimate from an instrumental variable analysis is a valid test statistic for the causal null hypothesis without requiring the assumptions of linearity, homogeneity, or monotonicity.^10^ In any case, the causal effect of intervention on a risk factor is likely to depend on several aspects of the intervention (e.g., its magnitude, duration, and pathway), and therefore will not precisely correspond to the estimate from a Mendelian randomization analysis.^11^ Hence, we would urge practitioners to view the assessment of causality as the primary result of a Mendelian randomization, and not to interpret any causal estimate too literally.^12^

We also assume that the genetic variants are mutually independent in their distributions, although extensions are available for most of the analysis methods in the case of correlated variants, provided that the correlation structure is known.^13^

Genetic variants are particularly suitable candidate instrumental variables, as they are fixed at conception, and hence cannot be affected by environmental factors that could otherwise lead to confounding or reverse causation.^14^ However, there are many well-documented ways in which the instrumental variable assumptions may be violated for any particular genetic variant, such as pleiotropy, linkage disequilibrium, and population stratification.^3,15^

For risk factors that are soluble protein biomarkers, there is often a gene region that encodes the protein (for example, the *CRP* gene region for C-reactive protein^16^), or a regulator or inhibitor of the protein (e.g., the *IL6R* gene region for interleukin-6^17^). Using one or more variants from such a gene region as instrumental variables would be ideal for a Mendelian randomization analysis, as these genetic variants would be the most likely to satisfy the instrumental variable assumptions, and the most informative proxies for intervention on the risk factor.^18^ However, such genetic variants do not exist for many risk factors.

The approach of using multiple genetic variants in different gene regions is particularly suitable for complex risk factors that are multifactorial and polygenic, such as body mass index,^19^ height,^20^ or blood pressure.^21^ Summarized data (in particular, beta-coefficients and standard errors) on genetic associations with the risk factor can be combined with summarized data on genetic associations with the outcome (that are often publicly available for download) to provide causal effect estimates, under the assumption that the genetic variants are all instrumental variables.^22,23^ Using multiple genetic variants increases the power of a Mendelian randomization investigation compared with an analysis based on a single variant.^24^ However, even if only one of the genetic variants is not a valid instrumental variable, the causal estimate based on all the variants from a conventional Mendelian randomization analysis will be biased and type 1 (false positive) error rates will be inflated.^25,26^

In this article, we describe a range of sensitivity analyses that either support or question the validity of causal inference from a Mendelian randomization analysis with multiple genetic variants. These sensitivity analyses will be useful for judging whether a causal conclusion from such an analysis is plausible or not. We focus on those sensitivity analyses that can be implemented using summarized data only. We consider approaches under two broad categories: methods for assessing the instrumental variable assumptions, and robust analysis methods that rely on a less stringent set of assumptions than a conventional Mendelian randomization analysis.

We illustrate the approaches using the example of estimating the causal effect of C-reactive protein (CRP) on coronary artery disease (CAD) risk using four genetic variants in the CRP gene region,^16^ and using 17 genetic variants (eTable A1; http://links.lww.com/EDE/B114) that have been shown to be associated with CRP at a genome-wide level of significance in a large meta-analysis—see eFigure in Ref. 27—beta-coefficients represent per allele associations with log-transformed CRP concentrations. Genetic associations with CAD risk were taken from the CARDIoGRAM consortium;^28^ beta-coefficients represent per allele log odds ratios for CAD risk. Ethical approval for the analyses using four genetic variants in the *CRP* gene region was granted by the Cambridgeshire ethics review committee; for the analyses using 17 genetic variants associated with CRP concentrations and with CAD risk, ethical approval was granted to the constituent studies by local institutional review boards.

For reference, the causal estimate based on the genetic variants in the *CRP* gene region is null (odds ratio: 1.00, 95% confidence interval: 0.90, 1.13 per 1-SD increase in CRP concentrations [equal to a 1.05-unit increase in log-transformed CRP or a 2.86-fold increase]), whereas the “causal” estimate using an inverse-variance weighted method based on the genome-wide significant variants (a less reliable approach)^22^ is negative (odds ratio: 0.87, 95% confidence interval: 0.79, 0.96 per 1-SD increase). Software code for performing the proposed sensitivity analyses is provided in eAppendix A.1 and A.2 (http://links.lww.com/EDE/B114).

## ASSESSING THE INSTRUMENTAL VARIABLE ASSUMPTIONS

The first set of approaches we consider are those to assess whether the instrumental variable assumptions are likely to be satisfied or not for a set of genetic variants. We consider in turn the assessment of the association with measured confounders, the exploitation of a natural experiment in the form of a gene–environment interaction, examination of a scatter plot combined with a heterogeneity test, and of a funnel plot combined with a test for directional pleiotropy.

### Use of Measured Covariates

**FIGURE 2.**
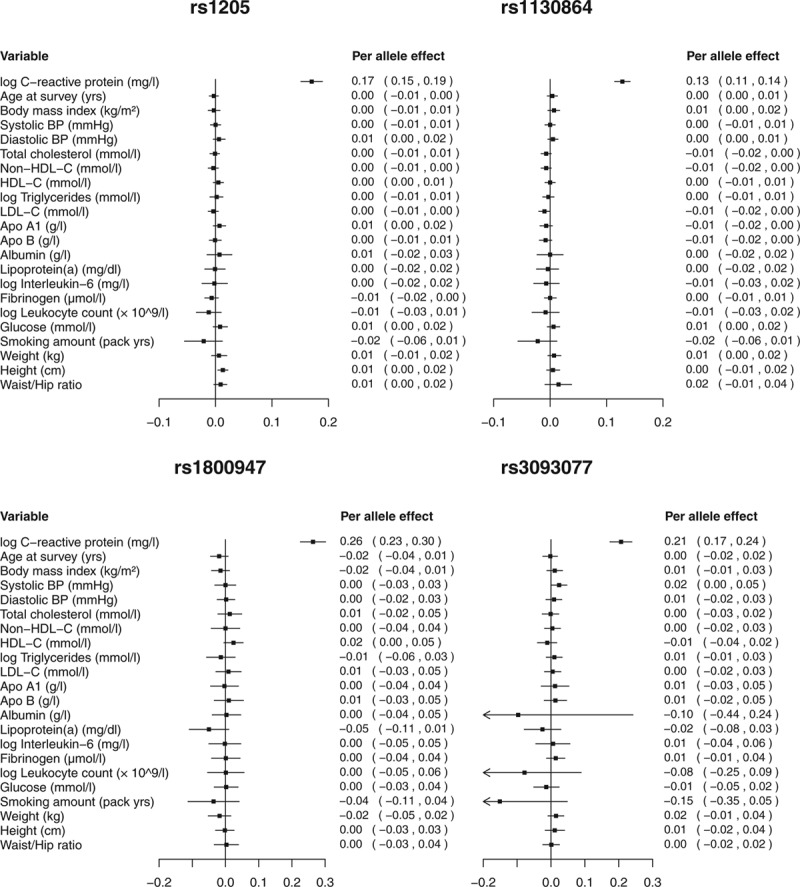
Associations (estimates in standard deviation units and 95% confidence intervals) of four genetic variants in the *CRP* gene region with a range of covariates per C-reactive protein increasing allele. Adapted from CRP CHD Genetics Collaboration.^16^

The assumption that an instrumental variable is not associated with confounders of the risk factor–outcome association is not fully testable, as not all confounders will be known or measured. However, the associations of genetic variants with measured covariates can be assessed. Lack of association of the instrumental variable with measured covariates does not imply lack of association with all confounders; however, an association with a measured covariate should be investigated carefully for a potential pleiotropic effect of the genetic variant. Figure 2, adapted from Wensley et al.,^16^ shows the associations of the four variants in the CRP gene region with a range of potential confounders. Associations are no stronger than would be expected by chance alone.

If there are covariates that by biological considerations should be downstream consequences of the risk factor, then the associations of genetic variants with these covariates can be assessed as positive controls to give confidence that the function of the genetic variants matches the known consequences of the risk factor. For instance, inhibition of interleukin-1 by the drug anakinra has been observed to lead to decreased levels of C-reactive protein and interleukin-6 in clinical trials. If genetic variants associated with interleukin-1 are also associated with both these covariates, this makes it more plausible that the variants are good proxies of intervention on interleukin-1 levels.^29^

**FIGURE 3.**
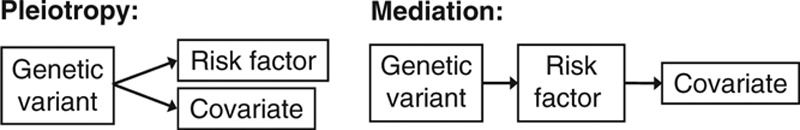
Diagram to illustrate the difference between pleiotropy (left, the association of the genetic variant with the covariate is independent of the risk factor) and mediation (right, the association of the genetic variant with the covariate is mediated entirely via the risk factor).

A benefit of the use of multiple genetic variants is the possibility to differentiate between pleiotropy and mediation, two mechanisms by which a genetic variant may be associated with a measured covariate (Figure 3). If a genetic variant is associated with a covariate independently of the risk factor (pleiotropy, or “horizontal pleiotropy”), then the instrumental variable assumptions are likely to be violated and the genetic variant should be excluded from an instrumental variable analysis, as the association with the covariate is likely to open a causal pathway from the variant to the outcome not via the risk factor. However, if the genetic variant is associated with a covariate due to its association with the risk factor of interest (mediation or “vertical pleiotropy”), and there is no alternative causal pathway from the variant to the outcome except for that via the risk factor, then the genetic variant is a valid instrumental variable.^23^

For instance, if increasing body mass index leads to increased blood pressure, then genetic variants that are instrumental variables for body mass index should also be associated with blood pressure. If multiple genetic variants that are candidate instrumental variables for body mass index are all concordantly associated with blood pressure, then it is plausible that the associations are due to mediation, not pleiotropy. In contrast, if only one or two variants are associated with blood pressure, then this is likely to be a manifestation of pleiotropy. Pleiotropy and mediation are not mutually exclusive (both could occur for the same covariate); however, this approach may give an insight into whether the association relates to a single genetic variant or to variants associated with the risk factor more widely.

In some cases, valid causal inference may still be possible even if a genetic variant has a pleiotropic association with a measured covariate; for instance, by adjusting for the covariate in the analysis model. However, if the Mendelian randomization investigation is performed using summarized data, then the investigator is unlikely to be able to adjust for covariates. An alternative approach with summarized data is a multivariable Mendelian randomization analysis, in which genetic associations with the outcome are regressed on the genetic associations with the risk factor and covariates in a multivariable weighted regression model.^30^

A practical difficulty of determining which variants to include in a Mendelian randomization analysis using measured covariates, aside from that of distinguishing between pleiotropy and mediation, is that of multiple testing. If there are large numbers of genetic variants and several measured covariates, then it is difficult to set a statistical significance threshold for rejecting a genetic variant as pleiotropic to balance between the desire to exclude invalid instrumental variables and the need to acknowledge the multiple tests. A sensible compromise is to consider multiple thresholds, for example, a conservative threshold to maximize robustness (a fixed threshold such as *P <* 0.01), and a liberal threshold to maximize power (such as a Bonferroni-corrected threshold taking into account the number of comparisons made).^23^ A similar approach was previously taken to assess the causal role of lipid fractions on CAD risk.^31^ If no causal effect is detected even in a liberal analysis, then the plausibility of a null causal finding increases.

### Gene–Environment Interaction

For some applications of Mendelian randomization, a further natural experiment may be available if the postulated causal effect is present in one stratum of the population, but absent in another.^32^ For example, the association of alcohol-related genetic variants with esophageal cancer risk is present in those who drink alcohol, but absent in abstainers.^33^ A gene–environment interaction provides evidence that a genetic association with the outcome in the population is a result of the risk factor; if it were a result of pleiotropy, then it would be likely to be present in both strata of the population. Gene–environment interactions may be difficult to find, but can provide convincing evidence of a causal effect.

One potential complication of such an analysis is the possibility of collider bias;^34^ by stratifying on the risk factor, associations between the genetic variants and the outcome may be distorted in the strata (in the examples above, in alcohol consumers/abstainers). To our knowledge, no systematic investigation has been conducted as to the degree that collider bias may lead to inappropriate causal inferences in a Mendelian randomization setting, although sensitivity analyses to assess the potential bias in the context of instrumental variable analysis with a single instrument are available.^35,36^

### Scatter Plot and Test for Heterogeneity

Even if the instrumental variable assumptions are in doubt for some or all of the variants, if several independent genetic variants in different gene regions are concordantly associated with the outcome, then a causal conclusion would seem reasonable.^37^ Although it is possible for the instrumental variable assumptions to be violated for all of the genetic variants, it is unlikely that pleiotropic effects for many different genetic variants would all result in the same direction of association with the outcome in the absence of an underlying causal effect of the risk factor.^38^ This is particularly true if there is a dose–response relationship in the per allele associations with the risk factor and with the outcome. An example of this is the relationship between low-density lipoprotein cholesterol (LDL-c) and CAD risk. See Figure 3 in Ref. 39. Genetic variants having considerably different magnitudes and mechanisms of association with LDL-c concentrations, including rare loss-of-function variants with substantial effect sizes, have proportional associations with CAD risk.

In a Mendelian randomization setting, a heterogeneity test is a statistical assessment of the compatibility of instrumental variable estimates based on individual genetic variants.^40^ In economics, this test is known as an over identification test, as the same causal effect is identified by each of the instrumental variables.^41^ Heterogeneity can be assessed visually by a scatter plot of the genetic associations with the outcome (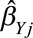 for genetic variant 
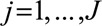
) against the genetic associations with the risk factor (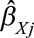), together with their confidence intervals. Each point on these graphs represents a genetic variant, and the points should be compatible with a straight line through the origin under the null. Any point that substantially deviates from this line should be investigated for potential pleiotropy.

**FIGURE 4.**
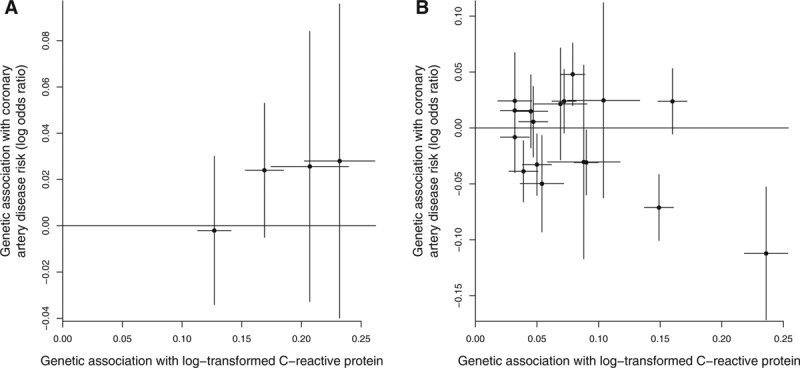
Scatter plots of genetic associations with the outcome against genetic associations with the risk factor (lines represent 95% confidence intervals) for Mendelian randomization analysis of CRP on coronary artery disease risk using genetic variants in the CRP gene region (left) and genetic variants throughout the genome (right) that have been demonstrated as associated with C-reactive protein at a genome-wide level of significance.

Scatter plots for the example of CRP and CAD risk are given in Figure 4; the plot using variants from the *CRP* gene region (left) demonstrates homogeneity of estimates, whereas the plot using genome-wide significant variants (right) demonstrates heterogeneity, with several clear outliers (although the genetic variants in the *CRP* gene region are partially correlated, so the homogeneity in the first case is somewhat artifactual).

A statistical test for heterogeneity can be performed using Cochran’s *Q* test on the causal estimates from each genetic variant 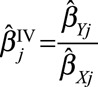, using the approximate standard errors 
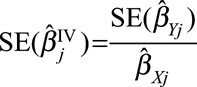
. This can be performed in standard statistical software packages for inverse-variance weighted meta-analysis. The statistic is calculated as


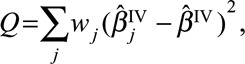


where 
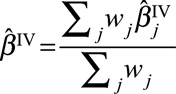
 is the (fixed-effect) inverse-variance weighted estimate based on all the genetic variants, and 
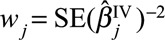
 are the inverse-variance weights. This statistic can be calculated using only summarized data. It should have a chi-squared distribution with 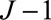 degrees of freedom under the null hypothesis of homogeneity. The amount of heterogeneity can also be expressed using the *I*^2^ statistic.^42^ Other heterogeneity tests include the Sargan test,^41^ which can be performed using individual-level data, or a likelihood ratio test using summarized data.^23^ An initial visual inspection for heterogeneity is important, as a formal statistical test may have low power particularly when there are few genetic variants.^43^ In the example of CRP and CAD risk, the *Q* statistic using genome-wide significant variants from across the genome is 71.9 (16 degrees of freedom, 
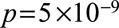
), indicating substantial heterogeneity.

The investigation of heterogeneity of causal estimates as an assessment of the instrumental variable assumptions relies on the assumption that all valid instrumental variables identify the same causal parameter. If not, then the heterogeneity test may over-reject the null.

### Funnel Plot and Test for Directional Pleiotropy

**FIGURE 5.**
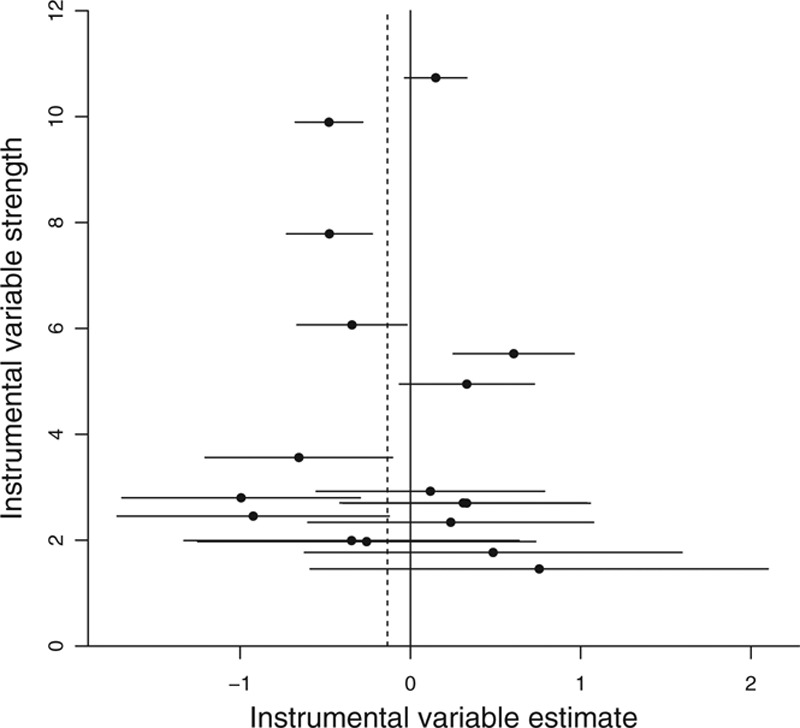
Funnel plot of instrument precision 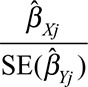 against instrumental variable estimates for each genetic variant separately 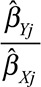 for Mendelian randomization analysis of C-reactive protein on coronary artery disease risk using genetic variants throughout the genome that have been demonstrated as associated with C-reactive protein at a genome-wide level of significance. Horizontal lines represent 95% confidence intervals for the instrumental variable estimates. Solid vertical line is at the null; dashed vertical line is the (fixed-effect) inverse-variance weighted estimate.

A funnel plot (taken from the meta-analysis literature^44^) of the instrumental variable precisions 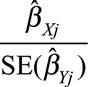 (the reciprocal of the standard error of the instrumental variable estimate) against the instrumental variable estimates 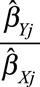 should be a symmetric funnel, in which more precise estimates are less variable. Any asymmetry in the funnel plot is a sign of directional pleiotropy (pleiotropic effects of genetic variants do not average to zero), meaning that causal estimates from the individual variants are biased on average. Although heterogeneity in causal estimates is concerning, provided that the pleiotropic effects of genetic variants are equally likely to be positive or negative, the overall causal estimate based on all the genetic variants may be unbiased. Directional pleiotropy is more serious, as it suggests that pleiotropic effects are not balanced, and thus that the overall causal estimate is biased. The funnel plot in the example of CRP on CAD risk for the genome-wide significant variants is shown in Figure 5. There is clear evidence of heterogeneity of causal effect estimates, but no evidence of departure from symmetry in this case.

Egger regression is a method for detecting small study bias (often interpreted as publication bias) in a meta-analysis of separate studies.^45^ The method can also be used for detecting directional pleiotropy from separate genetic variants.^46^ This can be implemented by a weighted regression of the genetic associations with the outcome (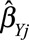) on the genetic associations with the risk factor (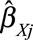) weighted by the inverse variance of the associations with the outcome (
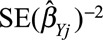
).^47^ The genetic associations should be orientated so that the associations with the risk factor all have the same sign. If there is no intercept term in this regression, the slope parameter is the inverse-variance weighted causal estimate.^48^ If there is an intercept term (as in Egger regression), then under the InSIDE assumption (see later), the intercept is the average pleiotropic effect of a genetic variant; if the intercept differs from zero, then there is evidence of directional pleiotropy.^46^ In the example of CRP on CAD risk for the genome-wide significant variants, the *P* value for the test of directional pleiotropy is 0.61, indicating no evidence of directional pleiotropy.

## ROBUST ANALYSIS METHODS

The second category of sensitivity analyses is that of robust analysis methods. Robust analysis methods allow different (and when the main purpose is to test the causal null hypothesis, weaker) assumptions than standard instrumental variable methods. In turn, we consider penalization methods, median-based methods, and Egger regression.

### Penalization Methods

We first consider methods in which the contribution of some genetic variants (e.g., heterogeneous or outlying variants) to the analysis is downweighted (or penalized). If the causal conclusion from a Mendelian randomization investigation depends only on a single genetic variant (particularly if the estimate from this variant is heterogeneous with those from other variants), then the result may be driven by a pleiotropic effect of that particular variant and not by the causal effect of the risk factor.

**FIGURE 6.**
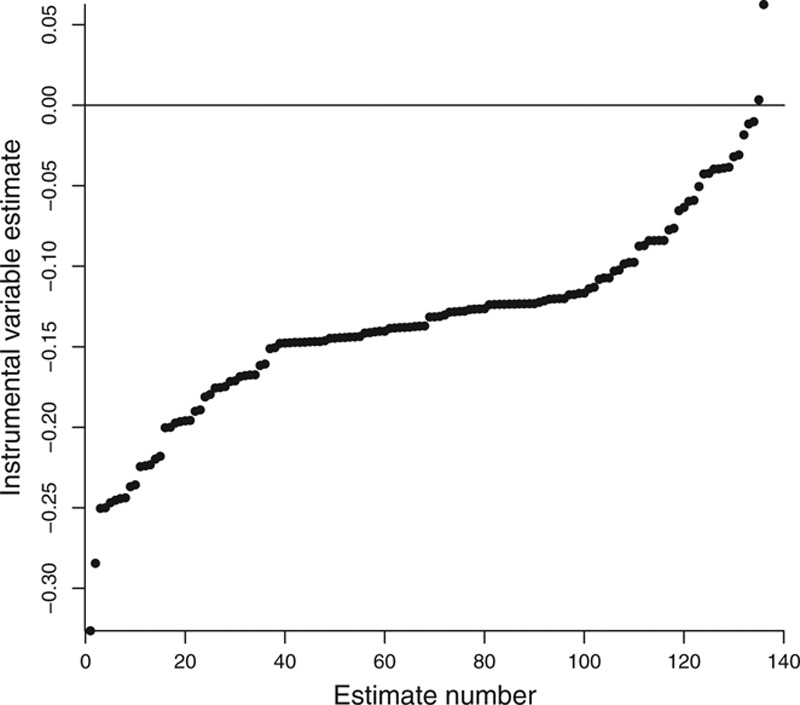
Estimates (ordered by magnitude) of causal effect of CRP on CAD risk from inverse-variance weighted method using 17 genome-wide significant genetic variants omitting variants systematically two at a time.

The simplest way of performing a penalization method is to omit some of the variants from the analysis. This could be done systematically. For example, with a small number of genetic variants, the causal estimates omitting one variant at a time could be considered. Alternatively, it could be done stochastically. For example, we could consider estimates omitting (say) 30% of the genetic variants at a time by selecting the 30% of variants at random a large number of times, and calculating the causal estimate in each case. This sensitivity analysis has been undertaken for the effect of LDL-c on aortic stenosis. See eFigure in Ref. 49. If the spread of results includes only (say) positive effect estimates, then we can be confident that the overall finding does not depend only on the influence of a few variants. However, even if only a small proportion of the estimates are discordant, these cases should be investigated and the omitted variants leading to the discordant estimates should be carefully investigated for potential violations of the instrumental variable assumptions. The causal estimates for the example of CRP on CAD risk based on the genome-wide significant variants using the inverse-variance weighted method are displayed in Figure 6. Two of the 17 variants are omitted from the analysis in turn in a systematic way, and then the 136 resulting estimates are arranged in order of magnitude. The overall estimate excluding the two strongest variants with negative causal estimates is positive, indicating that the overall negative finding based on all the variants seems to be driven by these two variants, and is not supported by the majority of variants.

A more focused approach to omitting genetic variants is to omit genetic variants from the analysis with heterogeneous instrumental variable estimates. This could be done by calculating the contribution to Cochran’s *Q* statistic for each genetic variant, and omitting any variant whose contribution to the statistic is greater than the upper 95th percentile of a chi-squared distribution on one degree of freedom (3.84). This approach has been applied for investigating the causal effect of lipid fractions on CAD risk.^50^ More formal penalization methods have been proposed using L1-penalization to downweight the contribution of outlying variants to the analysis in a continuous way.^51,52^ These methods have desirable theoretical properties, giving consistent estimates of the causal effect even if up to half of the genetic variants are not valid instrumental variables. However, they require individual-level data and a one-sample setting (genetic variants, risk factor, and outcome measurements are available for the same individuals).

### Median-based Methods

An alternative family of methods that gives consistent estimates when up to half the genetic variants are not valid instrumental variables, but that can be performed using summarized data rather than individual-level data, are median-based methods. If 50% or more of the genetic variants are valid instrumental variables, then the instrumental variable estimates for these variants will all be consistent estimates of the causal effect. In particular, this implies that the median of all the instrumental variable estimates based on the individual genetic variants will be a consistent estimate.^51^

However, the median estimate is likely to be inefficient, as the individual instrumental variable estimates from each genetic variant receive equal weight in the analysis. An alternative is to construct a weighted median estimate, defined as the median of an empirical distribution in which each instrumental variable estimate appears with probability proportional to the inverse of its variance.^53^ Then, more precise instrumental variable estimates receive more weight in the weighted median function. The weighted median estimate is consistent under the assumption that genetic variants representing over 50% of the weight in the analysis are valid instruments. This is a subtly different assumption to the assumption that over 50% of the genetic variants are valid instruments, although it is not clear that one or other of the assumptions is more plausible generally. Confidence intervals for the median and weighted estimates can be estimated using bootstrapping.

### Egger Regression

The Egger regression method was introduced above as a test for directional pleiotropy; this test does not make any assumption about the genetic variants. However, under an assumption that is weaker than standard instrumental variable assumptions, the slope coefficient from the Egger regression method provides an estimate of the causal effect that is consistent asymptotically even if all the genetic variants have pleiotropic effects on the outcome.^46^ This is the assumption that pleiotropic effects of genetic variants (i.e., direct effects of the genetic variants on the outcome that do not operate via the risk factor) are independent of instrument strength (known as the InSIDE assumption—Instrument Strength Independent of Direct Effect). This same assumption was considered by Kolesár et al.^54^ with individual-level data. The motivation for the Egger regression method is that, under the InSIDE assumption, stronger genetic variants should have more reliable estimates of the causal effect than weaker variants. Once the average pleiotropic effect of variants is accounted for through the intercept term in Egger regression, any residual dose–response relationship in the genetic associations provides evidence of a causal effect. The Egger regression estimate is consistent under the InSIDE assumption as the sample size tends to infinity if the correlation between the direct effects and instrument strength is exactly zero; otherwise it is consistent as the sample size and the number of genetic variants both tend to infinity. As previously stated, Egger regression assumes linearity and homogeneity in the associations between the genetic variants, risk factor, and outcome.

The InSIDE assumption may not be satisfied in practice, particularly if the pleiotropic effects of genetic variants on the outcome act via a single confounding variable. There is some evidence for the general plausibility of the InSIDE assumption, as associations of genetic variants with different phenotypic variables have been shown to be largely uncorrelated in an empirical study.^55^ The Egger regression estimate may have much wider confidence intervals than those from other methods in practice, as it relies on variants having different strengths of association with the risk factor. A situation with many independent genetic variants having identical magnitudes of association with the risk factor and with the outcome would intuitively provide strong evidence of a causal effect; however, the Egger estimate in this case would not be identified.

The Egger regression method gives consistent estimates if all the genetic variants are invalid instruments provided that the InSIDE assumption is satisfied, whereas the penalization and median-based methods rely on over half of the genetic variants being valid instrumental variables for consistent estimation. However, the penalization and median-based methods allow more general departures from the instrumental variable assumptions for the invalid instruments. In practice, it would seem prudent to compare estimates from a range of methods. If all methods provide similar estimates, then a causal effect is more plausible. For example, using genetic variants chosen solely on the basis of their association with the risk factor, a broad range of methods affirmed that LDL-c was a causal risk factor for CAD risk. However, the causal effect of HDL-c on CAD risk suggested by a liberal Mendelian randomization analysis using the inverse-variance weighted method (see also 31) was not supported by robust analysis methods.^53^ The median-based and Egger regression methods have also been shown to have lower type 1 (false positive) error rates than the inverse-variance weighted method in simulation studies with some invalid instrumental variables for finite sample sizes,^46,53^ although they were above the nominal level in the case of directional pleiotropy (for the median method), and when the InSIDE assumption was violated (for the Egger regression method).

### Example: C-reactive Protein and Coronary Artery Disease Risk

The robust methods described in this article were applied to the example of CRP and CAD risk using genome-wide significant variants; the code for performing these analyses is given in eAppendix A.3 (http://links.lww.com/EDE/B114). The inverse-variance weighted method was originally proposed as a fixed-effect meta-analysis of the causal estimates from each of the genetic variants.^21,22,48^ However, if there is heterogeneity between the causal estimates of different variants (as is the case here), a random-effects model would be more appropriate. In Egger regression, heterogeneity is expected as genetic variants that are not valid instrumental variables but satisfy the InSIDE assumption will give heterogeneous causal estimates. We consider fixed-effect and multiplicative random-effects models for both the inverse-variance weighted and Egger regression methods.^56^ Also, we consider simple (i.e., unweighted) median and weighted median estimates.

The fixed-effect inverse-variance weighted and Egger regression estimates suggest an inverse causal effect of CRP on CAD risk (Table 1). However, the corresponding random-effects analyses imply that there is no convincing evidence for a causal effect. Moreover, the simple median estimate is in the opposite direction. This arises because, although the strongest genetic variants have negative causal estimates, the majority of genetic variants have positive causal estimates. The inconsistency of the estimates from different methods indicates that the genome-wide significant variants for CRP are not all valid instrumental variables, and that a causal conclusion based on these variants would be unreliable.

**TABLE 1.**
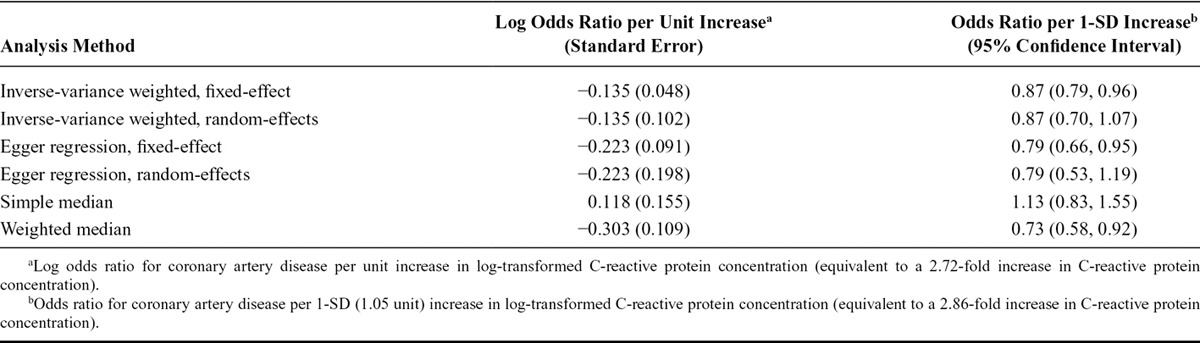
Estimates of Causal Effect of C-reactive Protein on Coronary Artery Disease Risk Based on 17 Genome-wide Significant Variants

## DISCUSSION

When multiple genetic variants from different gene regions are used in a Mendelian randomization analysis, it is highly implausible that all the genetic variants satisfy the instrumental variable assumptions. This does not preclude a causal conclusion; however, it means that a simple instrumental variable analysis alone should not be relied on to give a causal conclusion. Inappropriate and naive application of standard Mendelian randomization methods may lead to exactly the same problems of unmeasured confounding that the technique was designed to avoid.

In this article, we have discussed a range of sensitivity analyses that can be used to question the plausibility of a Mendelian randomization analysis using multiple variants, focusing on those analyses that are judged to be most useful to an applied analyst and those that can be performed using summarized data. The different approaches are summarized in Table 2. Not every sensitivity analysis may be appropriate for each case, but some effort should be made to investigate whether a causal finding is robust to violations of the instrumental variable assumptions.

**TABLE 2.**
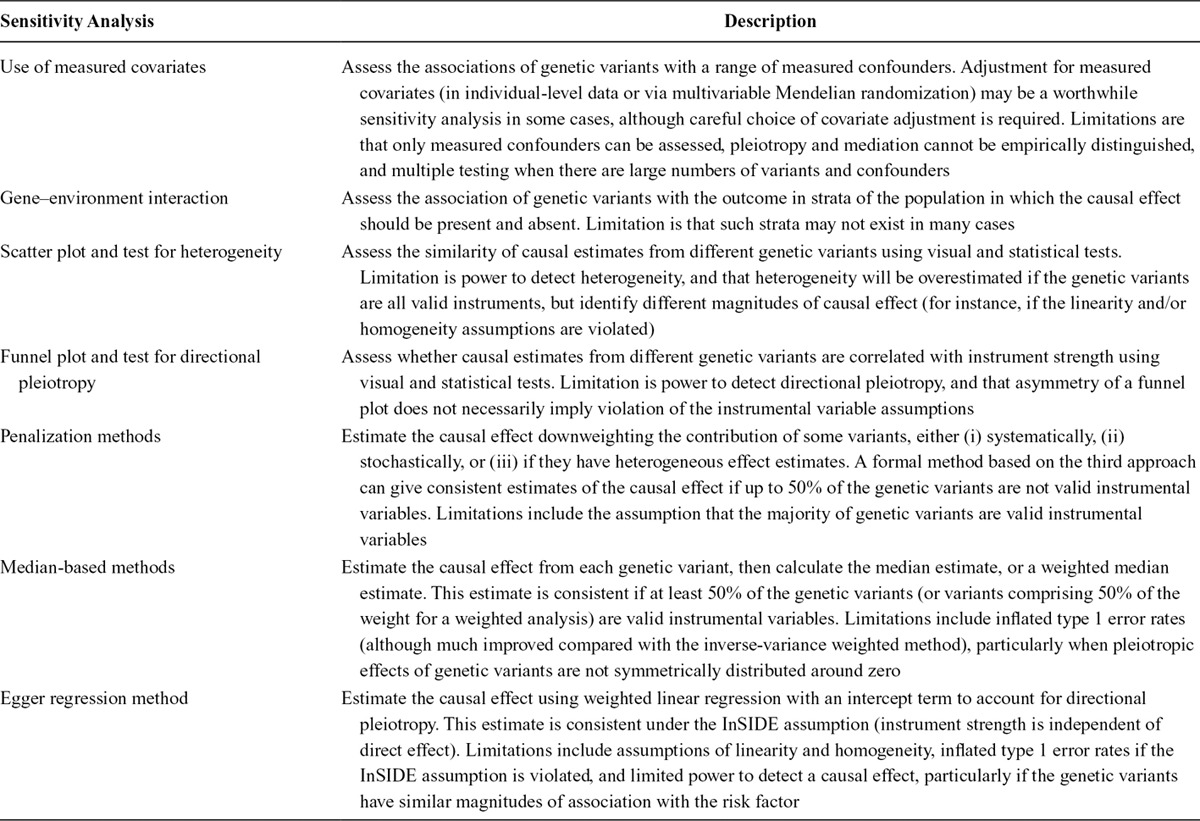
Summary of Sensitivity Analyses Considered in this Article, and Limitations of Each of the Proposed Analyses

### Comparison with Previous Literature

From its initial popularization, proponents of Mendelian randomization have been candid about the stringent and untestable assumptions required in Mendelian randomization.^3,14^ However, applied investigations have not always reflected this need for caution. In comparison with previous attempts to offer robust approaches for causal inference in Mendelian randomization, we have here repeated some of the guidance of Glymour et al.,^32^ specifically relating to the search for gene–environment interactions and to testing for heterogeneity between the estimates from different variants. We have not discussed the use of bounds for instrumental variable estimates^57^ (as these are usually uninformative in all but the most pathological cases, and cannot be calculated when the risk factor is continuous^5^), and the adjustment of gene–outcome associations for the risk factor. Substantial attenuation of the association on adjustment for the risk factor is expected if the genetic variant is a valid instrumental variable; however, such attenuation may not occur in practice, for example, due to measurement error in the exposure^58^—conversely, some attenuation may occur for an invalid instrumental variable. VanderWeele et al.^10^ suggest using Mendelian randomization as a test for a causal effect without providing an effect estimate, and provide a sensitivity analysis for a pleiotropic effect on an unmeasured confounder. However, this sensitivity analysis is only designed for use with a single genetic variant, so it cannot be applied in the majority of cases.

Much of the criticism of VanderWeele et al.^10^ over the precise definition of the causal parameter estimated in Mendelian randomization is warranted, although a response would be to have a less literal interpretation of effect estimates in Mendelian randomization and to view the primary finding from a Mendelian randomization investigation as the assessment of causation rather than the estimation of a causal effect. Violations of the assumptions of homogeneity and/or linearity of the causal effect would also lead to difficulties in interpreting the causal estimate, although they are unlikely to lead to inappropriate causal inferences or inflated type 1 error rates under the null.^59^ A causal estimate is useful to combine and compare evidence from multiple genetic variants, but it can be primarily interpreted as a test of the null hypothesis of no causal effect and only secondarily as a guide to the expected result of intervening on the risk factor in practice. As such, we regard violations of the instrumental variable assumptions necessary for valid causal inferences as first-order concerns, but violations of the assumptions necessary for the estimation of a causal effect as second-order concerns.

### Summarized Data and Two-sample Mendelian Randomization

Although the opportunities to assess the validity of genetic variants as instrumental variables are inherently less than if individual-level data were available, all of the sensitivity analyses discussed in this article can equally be performed using summarized data (although assessing associations with covariates may be difficult to do in a consistent way or in a consistent set of individuals, and summarized data for assessing a gene–environment interaction is unlikely to be routinely made available). A further concern with summarized data is the use of two-sample analyses, in which data on the gene–risk factor and gene–outcome associations are taken from nonoverlapping datasets.^60^ It is important in this case that the two samples are similar, particularly with regard to ethnic origin, as it is necessary for the instrumental variable assumptions to hold in both samples, as well as for estimates from each sample to be relevant to the other sample. This is not to discourage the use of summarized data or two-sample Mendelian randomization analyses, but to acknowledge that the bar for evidential quality is even higher in this case.

### Genetic Variants with Different Functional Effects

In this article, we have assumed that there is a single causal effect of the risk factor on the outcome, and interpreted deviation from this (i.e., heterogeneity of causal effect estimates) as evidence that the instrumental variable assumptions are violated for some of the genetic variants. In reality, if genetic variants have different functional effects on the risk factor, then different magnitudes of causal effect may be expected. For instance, genetic variants associated with body mass index may have different biological mechanisms giving rise to the association, and may affect the outcome to different extents. Heterogeneity between causal estimates based on sets of genetic variants grouped according to their biological function may help reveal which mechanisms are causal.^61^ Alternatively, different causal effects may arise under failure of the assumptions of homogeneity of the genetic association with the risk factor or linearity of the effect of the risk factor on the outcome. In this case, the causal estimates presented in this article still provide a valid test of the causal null hypothesis, but do not have an interpretation as estimates of a causal parameter.^12^

### Pleiotropy and Other Violations of the Instrumental Variable Assumptions

In this article, we have discussed violations of the instrumental variable assumptions primarily using the language of pleiotropy. Some other ways in which the instrumental variable assumptions may be violated (such as linkage disequilibrium with another functional variant) can also be expressed in terms of pleiotropy, and so these situations can be dealt with similarly. In particular, violations of the exclusion restriction assumption (i.e., no effect of the genetic variant on the outcome except for that via the risk factor) can be expressed as pleiotropic effects.^62^ A notable exception is population stratification, which can be best addressed by choice of study population (a population of uniform ethnicity should be used whenever possible). Population stratification is commonly addressed by the adjustment in the genetic association analyses for genome-wide principal components.^63^ While this adjustment has proved successful in some cases, it is not guaranteed to eliminate population stratification. Another potential source of bias that does not correspond to pleiotropy is selection bias, including sample ascertainment and informative censoring.^64^

Further potential problems for Mendelian randomization that have been identified include measurement error in the risk factor and multiple versions of the risk factor.^32^ Classical (nondifferential, zero mean) measurement error in the risk factor does not lead to bias in instrumental variable estimates.^65^ As the misspecification of weights in an allele score does not lead to inappropriate causal inferences,^25^ it is likely that any plausibly realistic pattern of measurement error would not lead to inflation of type 1 error rates under the null. If there are multiple versions of the risk factor, then this would lead to difficulties in interpreting the causal findings. For example, if body mass index is treated as the risk factor in the analysis, but in fact the true causal risk factor is abdominal obesity (or some other more specific measure of obesity), then the sensitivity analyses of this article would be appropriate for assessing the validity of a causal finding, assuming that the surrogate risk factor (here, body mass index) and the true causal risk factor (here, abdominal obesity) are correlated. However, they will not help to identify the specific causal risk factor; only biological knowledge can help here.

We expect the sensitivity analyses discussed in the article to be able to detect violations of the instrumental variable assumptions regardless of how these violations arise, although it is unlikely that the some consistency properties of the robust analysis methods (in particular the Egger regression method) will hold.

## CONCLUSIONS

The increasing size and coverage of genome-wide association studies and the increasing availability of summarized data on genetic associations are making the application of Mendelian randomization simpler. However, consideration must be given as to the robustness of findings to violations of the instrumental variable assumptions. Although no method can provide an infallible test of causation, the methods for sensitivity analysis described in this article will help to judge whether a causal conclusion from a Mendelian randomization analysis is reasonable or not. Aside from cases in which the selection of the genetic variants and their justification as instrumental variables is motivated by strong biological understanding, a Mendelian randomization analysis in which no assessment of the robustness of the findings has been made should be viewed as speculative.

**Key messages:**

- Mendelian randomization investigations are becoming more powerful and simpler to perform, due to the increasing size and coverage of genome-wide association studies and the increasing availability of summarized data on genetic associations with risk factors and disease outcomes.
- However, when using multiple genetic variants from different gene regions in a Mendelian randomization analysis, it is highly implausible that all the genetic variants satisfy the instrumental variable assumptions.
- This means that a simple instrumental variable analysis alone should not be relied on to give a causal conclusion.
- In this article, we discuss a range of sensitivity analyses that will either support or question the validity of causal inference from a Mendelian randomization analysis with multiple genetic variants.
- Aside from cases in which the justification of the instrumental variable assumptions is supported by strong biological understanding, a Mendelian randomization analysis in which no assessment of the robustness of the findings to violations of the instrumental variable assumptions has been made should be viewed as speculative and incomplete.
- In particular, Mendelian randomization investigations with large numbers of genetic variants without such sensitivity analyses should be treated with skepticism.

## Supplementary Material
