# Supplementary material for: Sensitivity Analyses for Robust Causal Inference from Mendelian Randomization Analyses with Multiple Genetic Variants
Source: Epidemiology. 2016 Nov 30;28(1):30–42. doi: 10.1097/EDE.0000000000000559 (PMC5133381; doi:10.1097/EDE.0000000000000559)
Supplement: Supplementary file 1 [file ede-28-030-s001.pdf]

Online supplemental material

A.1 Generic software code

We provide R code to implement the methods discussed in this paper. The associations of the genetic variants with the risk factor are denoted `betaXG` with standard errors `sebetaXG`. The associations of the genetic variants with the outcome are denoted `betaYG` with standard errors `sebetaYG`. We assume that the genetic variants are independently distributed.

Inverse-variance weighted estimate:

```
betaIVW           = sum(betaYG*betaXG*sebetaYG^-2)/sum(betaXG^2*sebetaYG^-2)
sebetaIVW.fixed  = 1/sqrt(sum(betaXG^2*sebetaYG^-2))
```

Equivalently, the inverse-variance weighted estimate can be calculated by weighted linear regression:

```
betaIVW           = summary(lm(betaYG~betaXG-1, weights=sebetaYG^-2))$coef[1]
sebetaIVW.fixed  = summary(lm(betaYG~betaXG-1, weights=sebetaYG^-2))$coef[1,2]/
                     summary(lm(betaYG~betaXG-1, weights=sebetaYG^-2))$sigma
sebetaIVW.random = summary(lm(betaYG~betaXG-1, weights=sebetaYG^-2))$coef[1,2]/
                max(summary(lm(betaYG~betaXG-1, weights=sebetaYG^-2))$sigma,1)
```

In the fixed-effect model, we divide the reported standard error by the estimated residual standard error, to fix the residual standard error to take the value 1.[56] In the random-effects model, we divide by the estimated residual standard error in the case of underdispersion (the variability in the genetic associations is less than would be expected by chance alone). But in the case of overdispersion (that is, heterogeneity of causal effect estimates), no correction is made. This is a multiplicative random-effects model. The point estimate is unaffected by the choice of a fixed- or random-effects model.

Scatter plot:

```
betaYG = betaYG*sign(betaXG); betaXG = abs(betaXG)
plot(betaXG, betaYG, xlim=c(min(betaXG-2*sebetaXG, 0), max(betaXG+2*sebetaXG)),
 ylim=c(min(betaYG-2*sebetaYG, 0), max(betaYG+2*sebetaYG, 0)))
for (j in 1:length(betaXG)) {
 lines(c(betaXG[j],betaXG[j]), c(betaYG[j]-1.96*sebetaYG[j], betaYG[j]+1.96*sebetaYG[j]))
 lines(c(betaXG[j]-1.96*sebetaXG[j],betaXG[j]+1.96*sebetaXG[j]), c(betaYG[j], betaYG[j]))
 }
abline(h=0, lwd=1); abline(v=0, lwd=1)
```

Test for heterogeneity:

```
metagen(betaYG/betaXG, sebetaYG/betaXG)
p.hetero = 1-pchisq(metagen(betaYG/betaXG, sebetaYG/betaXG)$Q,
   metagen(betaYG/betaXG, sebetaYG/betaXG)$df.Q)
```

We use the `metagen` command, from the package *meta*.

Funnel plot:

```
plot(betaYG/betaXG, betaXG/sebetaYG, xlim=c(min((betaYG-2*sebetaYG)/betaXG),
  max((betaYG+2*sebetaYG)/betaXG)), ylim=c(0, max(betaXG/sebetaYG)))
for (j in 1:length(betaXG)) {
 lines(c((betaYG[j]-1.96*sebetaYG[j])/betaXG[j],  (betaYG[j]+1.96*sebetaYG[j])/betaXG[j]),
  c(betaXG[j]/sebetaYG[j], betaXG[j]/sebetaYG[j]))
 }
abline(h=0, lwd=1); abline(v=0, lwd=1)
```

Test for directional pleiotropy (intercept test from Egger regression method):

```
betaYG = betaYG*sign(betaXG); betaXG = abs(betaXG)
interEGGER          = summary(lm(betaYG~betaXG, weights=sebetaYG^-2))$coef[1,1]
seinterEGGER.fixed  = summary(lm(betaYG~betaXG, weights=sebetaYG^-2))$coef[1,2]/
                      summary(lm(betaYG~betaXG, weights=sebetaYG^-2))$sigma
seinterEGGER.random = summary(lm(betaYG~betaXG, weights=sebetaYG^-2))$coef[1,2]/
                  min(summary(lm(betaYG~betaXG, weights=sebetaYG^-2))$sigma, 1)
p.dpleio.fixed      = 2*(1-pt(abs(interEGGER/seinterEGGER.fixed), df=length(betaXG)-2))
p.dpleio.random     = 2*(1-pt(abs(interEGGER/seinterEGGER.random),df=length(betaXG)-2))
```

The `p.dpleio` variables are the p-values for the test of directional pleiotropy. The choice of fixed- or random-effects models is the same as that for the inverse-variance weighted method.

Median-based methods:

```
weighted.median <- function(betaIV.in, weights.in) {
  betaIV.order  = betaIV.in[order(betaIV.in)]
  weights.order = weights.in[order(betaIV.in)]
  weights.sum   = cumsum(weights.order)-0.5*weights.order
  weights.sum   = weights.sum/sum(weights.order)
        below   = max(which(weights.sum<0.5))
  weighted.est  = betaIV.order[below] + (betaIV.order[below+1]-betaIV.order[below])*
                  (0.5-weights.sum[below])/(weights.sum[below+1]-weights.sum[below])
  return(weighted.est) }

weighted.median.boot = function(betaXG.in, betaYG.in, sebetaXG.in, sebetaYG.in, weights.in){
 # the standard error is estimated based on 1000 bootstrap samples
med = NULL
for(i in 1:1000){
 betaXG.boot = rnorm(length(betaXG.in), mean=betaXG.in, sd=sebetaXG.in)
 betaYG.boot = rnorm(length(betaYG.in), mean=betaYG.in, sd=sebetaYG.in)
 betaIV.boot  = betaYG.boot/betaXG.boot
 med[i] = weighted.median(betaIV.boot, weights.in)
 }
return(sd(med)) }

betaIV             = betaYG/betaXG
weights            = rep(1, length(betaXG))
betaSIMPLEMED      = weighted.median(betaIV, weights)
sebetaSIMPLEMED    = weighted.median.boot(betaXG, betaYG, sebetaXG, sebetaYG, weights)

betaIV             = betaYG/betaXG
weights            = (sebetaYG/betaXG)^-2
betaWEIGHTEDMED    = weighted.median(betaIV, weights)
sebetaWEIGHTEDMED = weighted.median.boot(betaXG, betaYG, sebetaXG, sebetaYG, weights)
```

Egger regression method:

```
betaYG = betaYG*sign(betaXG); betaXG = abs(betaXG)
betaEGGER          = summary(lm(betaYG~betaXG, weights=sebetaYG^-2))$coef[2,1]
```

```
sebetaEGGER.fixed  = summary(lm(betaYG~betaXG, weights=sebetaYG^-2))$coef[2,2]/
                      summary(lm(betaYG~betaXG, weights=sebetaYG^-2))$sigma
sebetaEGGER.random = summary(lm(betaYG~betaXG, weights=sebetaYG^-2))$coef[2,2]/
                  min(summary(lm(betaYG~betaXG, weights=sebetaYG^-2))$sigma, 1)
```

A.2 Software code for example: C-reactive protein and coronary artery disease

Code for running example analyses in R:

```
betaXG  =c(0.160, 0.236, 0.149, 0.09, 0.079, 0.072, 0.047, 0.05, 0.069,
  0.039, 0.088, 0.032, 0.104, 0.045, 0.054, 0.032, 0.032)
betaYG  =c(0.0237903, -0.1121942, -0.0711906, -0.030848, 0.0479207, 0.0238895,
  0.005528, -0.0327605, 0.0214852, -0.0387675, -0.0304042, -0.0082261,
  0.0246432, 0.0148795, -0.0498487, 0.0155667, 0.0242003)
sebetaXG=c(0.006, 0.009, 0.006, 0.005, 0.005, 0.005, 0.006, 0.006, 0.011,
  0.006, 0.015, 0.006, 0.015, 0.007, 0.009, 0.006, 0.007)
sebetaYG=c(0.0149064, 0.0303084, 0.0150552, 0.0148339, 0.0143077, 0.0145478,
  0.0160765, 0.0140347, 0.0255237, 0.0139256, 0.0441698, 0.0162031,
  0.0444987, 0.016674, 0.0220043, 0.018098, 0.0219547)

weighted.median <- function(betaIV.in, weights.in) {
  betaIV.order  = betaIV.in[order(betaIV.in)]
  weights.order = weights.in[order(betaIV.in)]
  weights.sum   = cumsum(weights.order)-0.5*weights.order
  weights.sum   = weights.sum/sum(weights.order)
        below   = max(which(weights.sum<0.5))
  weighted.est  = betaIV.order[below] + (betaIV.order[below+1]-betaIV.order[below])*
                  (0.5-weights.sum[below])/(weights.sum[below+1]-weights.sum[below])
  return(weighted.est) }

weighted.median.boot = function(betaXG.in, betaYG.in, sebetaXG.in, sebetaYG.in, weights.in){
med = NULL
for(i in 1:1000){
 betaXG.boot = rnorm(length(betaXG.in), mean=betaXG.in, sd=sebetaXG.in)
 betaYG.boot = rnorm(length(betaYG.in), mean=betaYG.in, sd=sebetaYG.in)
 betaIV.boot  = betaYG.boot/betaXG.boot
 med[i] = weighted.median(betaIV.boot, weights.in)
 }
return(sd(med)) }

betaIVW            = summary(lm(betaYG~betaXG-1, weights=sebetaYG^-2))$coef[1]
sebetaIVW.fixed    = summary(lm(betaYG~betaXG-1, weights=sebetaYG^-2))$coef[1,2]/
                       summary(lm(betaYG~betaXG-1, weights=sebetaYG^-2))$sigma
sebetaIVW.random   = summary(lm(betaYG~betaXG-1, weights=sebetaYG^-2))$coef[1,2]/
                  min(summary(lm(betaYG~betaXG-1, weights=sebetaYG^-2))$sigma,1)

betaYG = betaYG*sign(betaXG); betaXG = abs(betaXG)
betaEGGER          = summary(lm(betaYG~betaXG, weights=sebetaYG^-2))$coef[2,1]
sebetaEGGER.fixed  = summary(lm(betaYG~betaXG, weights=sebetaYG^-2))$coef[2,2]/
                       summary(lm(betaYG~betaXG, weights=sebetaYG^-2))$sigma
sebetaEGGER.random = summary(lm(betaYG~betaXG, weights=sebetaYG^-2))$coef[2,2]/
                  min(summary(lm(betaYG~betaXG, weights=sebetaYG^-2))$sigma, 1)

betaIV             = betaYG/betaXG
weights            = rep(1, length(betaXG))
betaSIMPLEMED      = weighted.median(betaIV, weights)
sebetaSIMPLEMED    = weighted.median.boot(betaXG, betaYG, sebetaXG, sebetaYG, weights)

betaIV             = betaYG/betaXG
weights            = (sebetaYG/betaXG)^-2
betaWEIGHTEDMED    = weighted.median(betaIV, weights)
sebetaWEIGHTEDMED  = weighted.median.boot(betaXG, betaYG, sebetaXG, sebetaYG, weights)
```

Output from the above code:

```
> summary(lm(betaYG~betaXG-1, weights=sebetaYG^-2))
Call:
```

```
lm(formula = betaYG ~ betaXG - 1, weights = sebetaYG^-2)
Coefficients:
       Estimate Std. Error t value Pr(>|t|)
betaXG  -0.1347     0.1018  -1.323    0.204
Residual standard error: 2.12 on 16 degrees of freedom
Multiple R-squared:  0.09859,   Adjusted R-squared:  0.04226
F-statistic:  1.75 on 1 and 16 DF,  p-value: 0.2045
> betaIVW
[1] -0.1346602
> sebetaIVW.fixed
[1] 0.04800911
> sebetaIVW.random
[1] 0.04800911

> summary(lm(betaYG~betaXG, weights=sebetaYG^-2))
Call:
lm(formula = betaYG ~ betaXG, weights = sebetaYG^-2)
Coefficients:
             Estimate Std. Error t value Pr(>|t|)
(Intercept)  0.009189   0.017517   0.525    0.608
betaXG      -0.222959   0.197960  -1.126    0.278
Residual standard error: 2.17 on 15 degrees of freedom
Multiple R-squared:  0.07797,   Adjusted R-squared:  0.01651
F-statistic: 1.269 on 1 and 15 DF,  p-value: 0.2777
> betaEGGER
[1] -0.2229592
> sebetaEGGER.fixed
[1] 0.09122585
> sebetaEGGER.random
[1] 0.1979596

> betaSIMPLEMED
[1] 0.117617
> sebetaSIMPLEMED
[1] 0.1535546

> betaWEIGHTEDMED
[1] -0.3031061
> sebetaWEIGHTEDMED
[1] 0.106813
```

A.3 Details of genome-wide significant genetic variants

eTable A1 provides information about the 17 genome-wide significant variants used in the example analysis of this paper for the 'causal' effect of C-reactive protein (CRP) on coronary artery disease (CAD) risk. Details of these variants are given, including the beta-coefficients and standard errors for their associations with CRP (log-transformed) and CAD risk (log odds ratios), together with the causal estimates based on each of these variants (log odds ratios for CAD per unit increase in log-transformed CRP).

rsid	Nearest gene	Effect allele	Association with CRP Beta (SE)	Association with CAD risk Beta (SE)	Causal estimate Estimate (SE)
rs2794520	*CRP*	C	0.160 (0.006)	0.024 (0.015)	0.149 (0.093)
rs4420638	*APOC1*	A	0.236 (0.009)	-0.112 (0.030)	-0.475 (0.128)
rs1183910	*HNF1A*	G	0.149 (0.006)	-0.071 (0.015)	-0.478 (0.101)
rs4420065	*LEPR*	C	0.090 (0.005)	-0.031 (0.015)	-0.343 (0.165)
rs4129267	*IL6R*	C	0.079 (0.005)	0.048 (0.014)	0.607 (0.181)
rs1260326	*GCKR*	T	0.072 (0.005)	0.024 (0.015)	0.332 (0.202)
rs12239046	*NLRP3*	C	0.047 (0.006)	0.006 (0.016)	0.118 (0.342)
rs6734238	*IL1F10*	G	0.050 (0.006)	-0.033 (0.014)	-0.655 (0.281)
rs9987289	*PPP1R3B*	A	0.069 (0.011)	0.021 (0.026)	0.311 (0.370)
rs10745954	*ASCL1*	A	0.039 (0.006)	-0.039 (0.014)	-0.994 (0.357)
rs1800961	*HNF4A*	C	0.088 (0.015)	-0.030 (0.044)	-0.346 (0.502)
rs340029	*RORA*	T	0.032 (0.006)	-0.008 (0.016)	-0.257 (0.506)
rs10521222	*SALL1*	C	0.104 (0.015)	0.025 (0.044)	0.237 (0.428)
rs12037222	*PABPC4*	A	0.045 (0.007)	0.015 (0.017)	0.331 (0.371)
rs13233571	*BCL7B*	C	0.054 (0.009)	-0.050 (0.022)	-0.923 (0.407)
rs2836878	*PSMG1*	G	0.032 (0.006)	0.016 (0.018)	0.486 (0.566)
rs4903031	*RGS6*	G	0.032 (0.007)	0.024 (0.022)	0.756 (0.686)

eTable A1: Details of genetic variants, beta-coefficients (standard errors, SE) for associations with C-reactive protein (CRP) taken from Dehghan et al.[27] and with coronary artery disease (CAD) risk taken from CARDIoGRAM consortium,[28] and causal effect estimates for 17 genome-wide significant variants.
